# Case report: left atrial Myxoma causing elevated C-reactive protein, fatigue and fever, with literature review

**DOI:** 10.1186/s12872-020-01397-1

**Published:** 2020-03-05

**Authors:** Jake Cho, Steven Quach, Justin Reed, Omeni Osian

**Affiliations:** 1 University of Central Florida College of Medicine, Graduate Medical Education, Orlando, FL 32827 USA; 2 HCA Ocala Regional Medical Center, Internal Medicine Residency Program, 1431 SW 1st Ave, Ocala, FL 34471 USA; 3 HCA Ocala Regional Medical Center, Internal Medicine Faculty, Ocala, FL 34471 USA; 4 HCA Ocala Regional Medical Center, General Surgery Residency Program Faculty, Ocala, FL 34471 USA; 5 HCA Ocala Regional Medical Center, Thoracic and Cardiac Surgery, Ocala, FL 34471 USA

**Keywords:** Myxoma, Fever, Cardiac mass, Stroke, Cardiothoracic surgery, Carney complex

## Abstract

**Background:**

A cardiac myxoma in a young person may pose a diagnostic challenge as symptoms may be variable and the differential diagnosis is wide. The differential diagnosis can include rheumatic mitral valve disease, pulmonary hypertension, endocarditis, myocarditis and vasculitis.

**Case presentation:**

This case report involves a 49 years old female with a 2.8 cm × 3.4 cm myxoma in the left atrium causing mitral valve obstruction. She presented with fatigue, fever of unknown origin, transient ischemic attack and shortness of breath. Prompt surgery is often recommended due to the risk of embolic complications or complete obstruction. Due to her symptoms, patient underwent successful cardiothoracic surgery to excise the myxoma within 2 weeks of confirmation by cardiac echocardiography.

**Conclusion:**

This case also emphasizes the diagnostic challenge as symptoms may be variable, ranging from fatigue, fever and shortness of breath to transient ischemic attack and at worst, sudden cardiac death. In conclusion, if a cardiac mass is suspected, echocardiography should be performed early. Surgical resection is curative and recurrence rate is very rare in sporadic isolated myxomas, however, recurrence can be higher in genetic diseases associated with increased frequency of myxomas such as Carney complex. This subpopulation of patients may present further research opportunity to learn more about the perioperative management of patients with myxomas such as determining the optimal time to surgical intervention and decision to anticoagulate.

## Learning objectives

This case report involves a 49 years old female with a myxoma in the left atrium causing mitral valve obstruction. She presented with fatigue, fever of unknown origin, transient ischemic attack and shortness of breath. Prompt surgery is often recommended due to the risk of embolic complications or complete obstruction. Due to her symptoms, patient underwent successful cardiothoracic surgery to excise the myxoma. Although the surgery was a success, this case emphasizes 3 main points. First, a myxoma in a young person may pose a diagnostic challenge as symptoms may be variable and the differential diagnosis is wide. The second point is that the cardiac mass in her case was unique in terms of size. The excised specimen was measured as 6.2 × 4.3 × 2.9 cm which would cause significant adverse hemodynamic effects on the heart. The third point is the emphasis on diagnostic echocardiography when a myxoma is suspected or if other diagnostic workup has been negative.

## Background

Primary cardiac tumors are rare, with an incidence of 1.38 per 100,000 people per year [1]. Cardiac myxomas are noncancerous primary tumors of the heart and constitute about 50% of all primary heart tumors. Myxomas occur most often in patients aged 30 to 70, predominantly in women [2]. About 75 to 85% of myxomas occur in the left atrium, 10 to 20% in the right atrium, and 5% in the ventricles [3]. Although patients can be asymptomatic, symptoms may include dyspnea, angina, syncope, cough, vertigo, fatigue, fever and stroke [4]. This case involves a patient with intermittent fevers, fatigue, and elevated C-reactive protein (CRP) that was eventually attributed to a myxoma.

## Case presentation

**Fig. 1:**
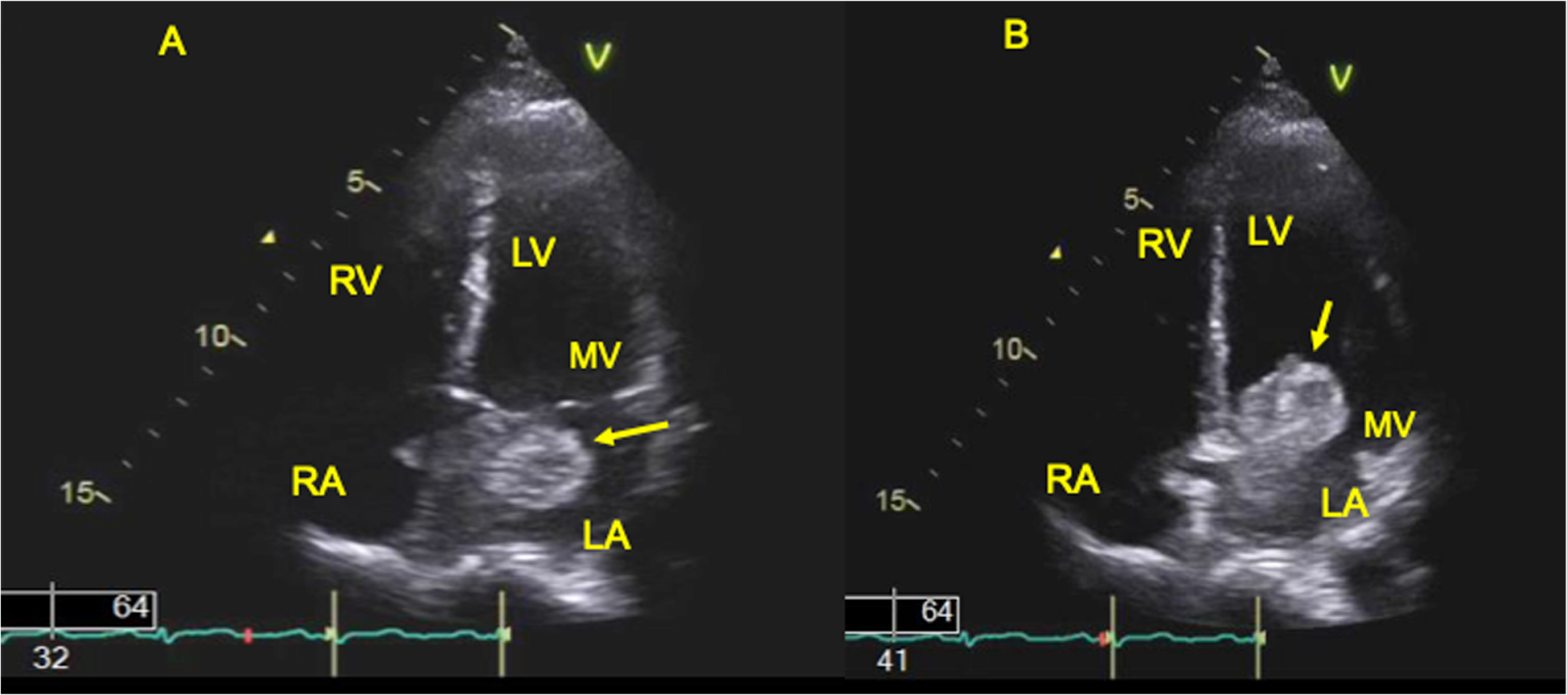
**a**: Transthoracic echocardiogram (TTE) apical view shows a large left atrial mass at the level of the mitral valve during systole (arrow). Abbreviations: LA-left atrium, LV-left ventricle, RA-right atrium, RV-right ventricle, MV-mitral valve. **b**: Transthoracic echocardiogram (TTE) apical view, showing the mass at the mitral valve prolapsing into the left ventricle during the diastolic cardiac cycle (arrow). Abbreviations: LA-left atrium, LV-left ventricle, RA-right atrium, RV-right ventricle, MV-mitral valve

The patient is a 49 years old female, with no significant medical history, who started experiencing symptoms of reduced exercise tolerance and easy fatigability along with increased vulnerability to viral illnesses that started about 1 year ago. She started having intermittent fevers of 100.5 °F with cough and rhinorrhea which was diagnosed as bronchitis and she was prescribed azithromycin. She also developed abdominal cramping and diarrhea and her CRP at that time was found to be elevated to 40.6 mg/L (normal < 10 mg/L). Abdominal CT did not show any abnormalities. The gastrointestinal symptoms and viral illness gradually resolved over the next month. A few months later, however, she suffered stroke like symptoms while at work and she was admitted for vertigo and presyncope. Her National Institutes of Health stroke scale score was 0 and Computed Tomography (CT) scan of her brain was negative for acute pathology. She was diagnosed with peripheral vertigo. Throughout the year, she continued to have fatigue and reported a 10 lbs. weight loss. Her CRP also continued to be elevated, reaching a zenith of 129 mg/L (normal < 10 mg/L). At this time, her fatigue, shortness of breath and heat intolerance became more pronounced. She continued to experience intermittent fevers with night sweats for about 3 weeks, prompting an infectious disease workup. The Infectious Disease specialist started her on Cefuroxime for 5 days, which did not help, and then switched her to Doxycycline for 10 days with inconclusive results. CT chest with contrast was ordered but unfortunately, she was unable to obtain the imaging due to delays with her health insurance. She was then referred to a pulmonologist to evaluate for possible pulmonary etiology. The pulmonologist was able to order the CT chest but imaging was obtained without contrast which did not identify any pathology such as pneumonia (Fig. 4a, b). Despite extensive workup including consultations from Infectious Disease and Pulmonology, a diagnosis was not reached to account for her constitutional symptoms of intermittent fevers, fatigue, dyspnea on exertion and elevated CRP. Over the next month, she experienced chest heaviness, “like a brick” in her chest along with severe limitations to physical activities. She was subsequently seen by her cardiologist in the clinic where her vital signs included a blood pressure of 115/72 mmHg, heart rate of 87 bpm, respiratory rate of 16 breaths per minute and a body mass index of 20.4. Physical exam was only positive for a 1/6 holosystolic murmur at the left sternal border radiating to the precordium. There was no neck vein distention and carotid artery auscultation did not reveal any bruits. There was no peripheral edema, cyanosis or clubbing. Pedal pulses and radial pulses were normal bilaterally. The lungs were clear to auscultation bilaterally, head and neck exam was negative for rhinorea, adenopathy or scleral conjunctivitis. Abdominal exam was benign with normal bowel sounds. Gait was normal and overall she appeared well developed and nourished but fatigued. Electrocardiogram (ECG) showed normal sinus rhythm with no ischemic changes (Fig. 5a to c), however, the transthoracic echocardiogram (TTE) showed left ventricular ejection fraction of 55–60% (LVEF) and a mass in the left atrium estimated as 2.8 × 3.4 cm with evidence of prolapse into the left ventricle during diastole (Fig. 1a, b).

### Clinical course

**Fig. 2:**
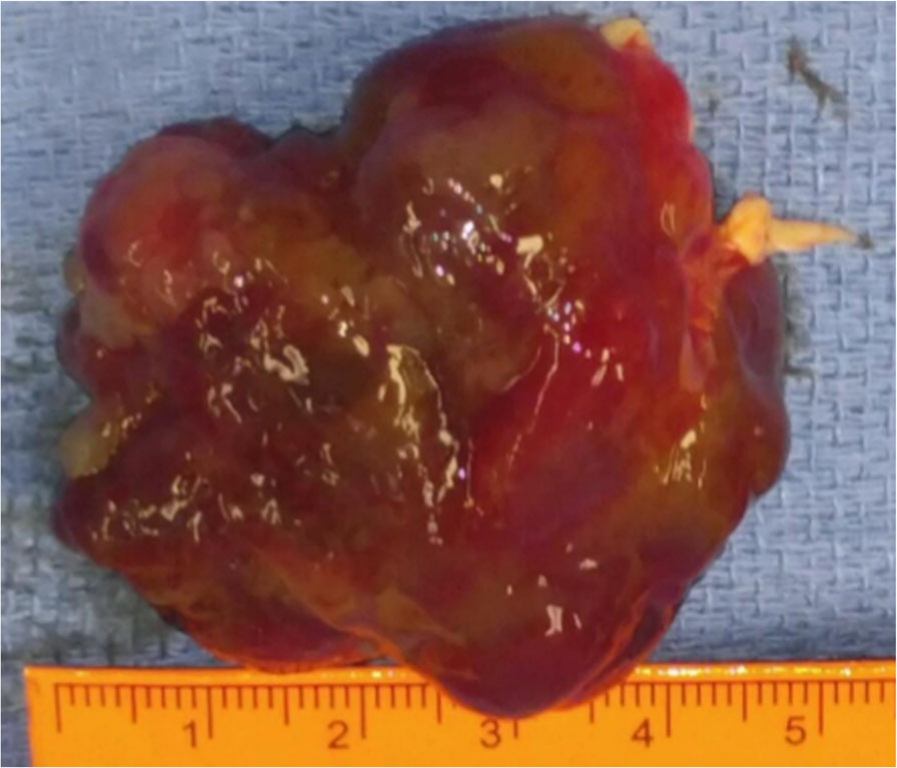
Post resection gross specimen with estimated size of 5.0 × 4.0 × 3.0 cm (ruler). Reported as 6.2 × 4.3 × 2.9 cm in the pathology report

**Fig. 3:**
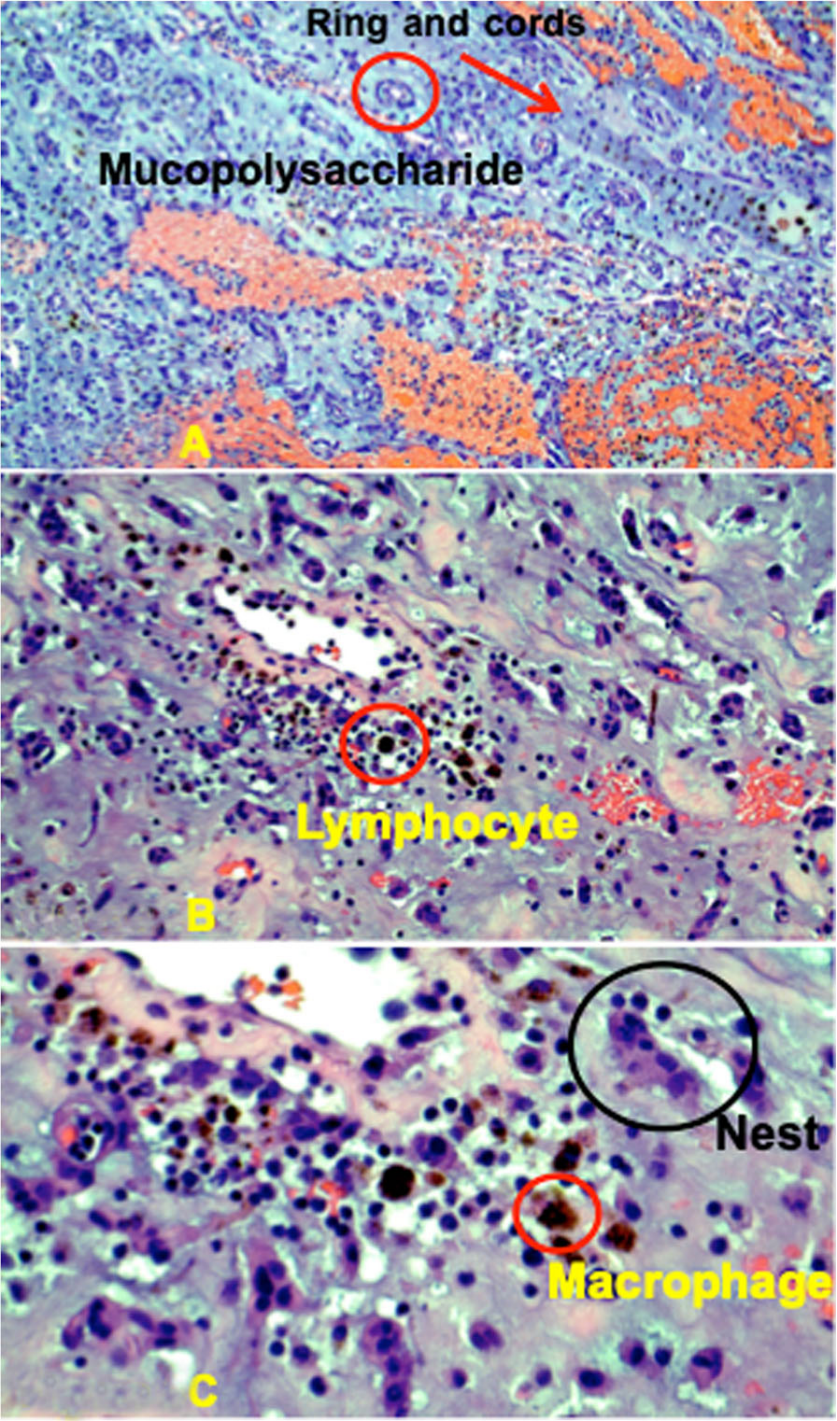
**a** Microscopy 10X magnification, structures of blue rings (red circle) and cords (red arrow), characteristic histologic features of myxomas. Alcian Blue Periodic Acid Schiff blue stain for mucopolysaccharide myxoid substance containing chondroitin sulfate and hyaluronic acid. **b:** Microscopy 20X magnification, lymphocytes (red circle), no mitotic Figs. **c:** Microscopy 40X magnification, hemosiderin laden macrophages (red circle), nests of myxoma cells (black circle), negative for malignancy

Given the symptoms, location and size of the mass, cardiac myxoma was suspected and Cardiothoracic Surgery was consulted. The patient was seen in the surgery clinic and agreed to surgical resection via sternotomy. During surgery, the patient was placed on cardiopulmonary bypass to perform a right atriotomy and incision at the fossa ovalis to access the left atrium. The mass was found to be attached to the left atrial septum via a stalk at the level of the fossa ovalis. Due to the significant size, a wider incision of the fossa ovalis was made to excise the myxoma along with the stalk and ensure clear margins. Although the size was estimated as 2.8 cm × 3.4 cm on TTE, the specimen was reported as 6.2 × 4.3 × 2.9 cm in the pathology report and described as an irregular shaped portion of translucent to dark tan-gray, soft, gelatinized mass tissue with overall smooth surfaces. A picture of the gross surgical specimen is shown in Fig. 2 with a visual estimation of the size. A bovine pericardial patch was used to reconstruct the septum and the right atriotomy was closed. The patient was subsequently weaned from cardiopulmonary bypass and two chest tube drains were placed within the pericardial space. The patient tolerated the procedure well and had an uneventful post-operative course. Histological evaluation of the specimen showed mostly matrix composed of an acid-mucopolysaccharide-rich stroma consistent with a myxoma but negative for malignancy (Fig. 3a to c). Post-operative EKG showed normal sinus rhythm. Post-operative TTE showed LVEF of 50% with no residual mass in the left atrium, normal mitral valve, no patent foramen ovale and no pericardial effusion.

### Patient perspective

**Fig. 4:**
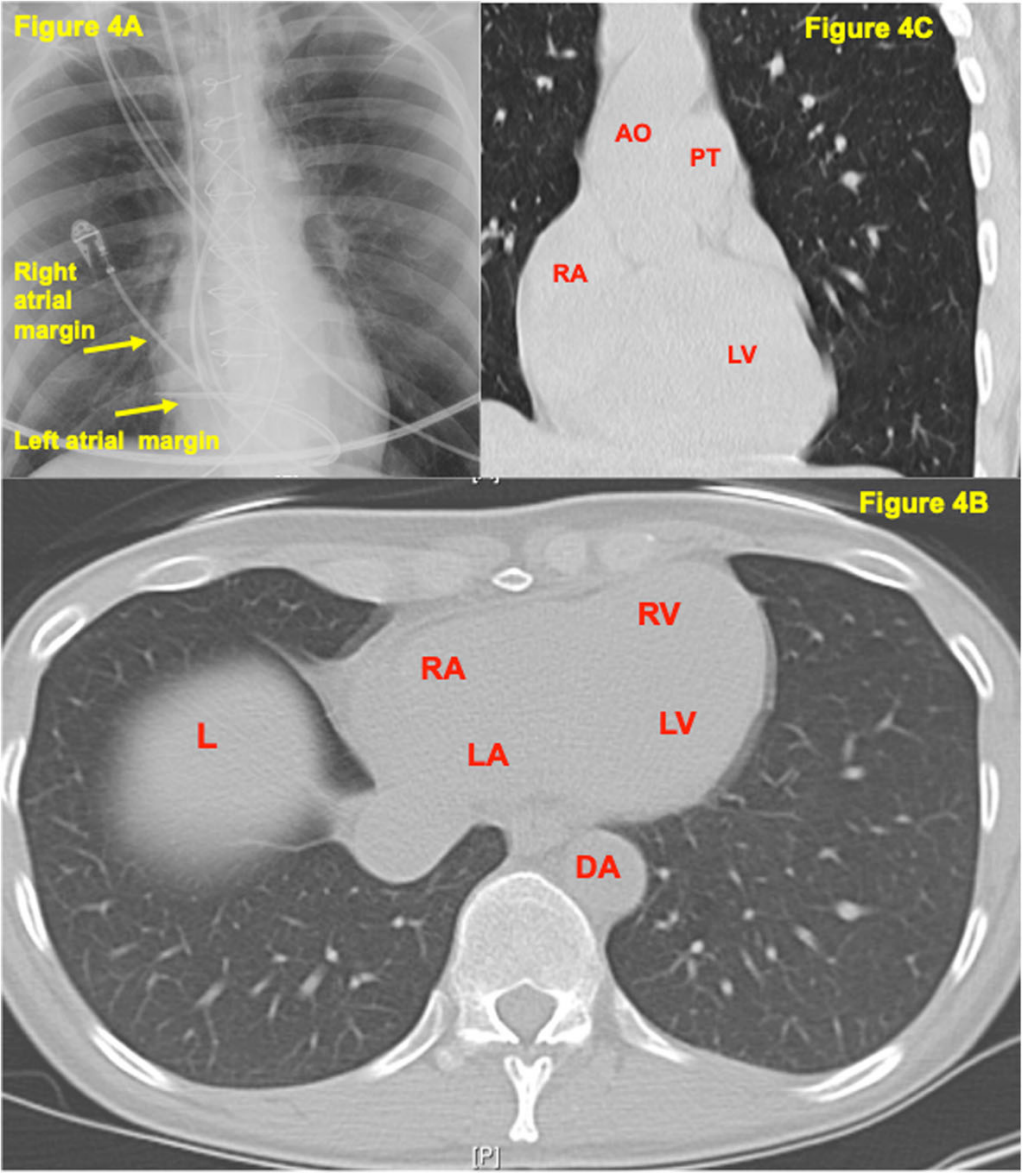
**a:** Peri-operative chest xray shows “double density” behind the right heart border, a common indication of left atrial enlargement. **b:** Pre-operative CT chest without contrast, axial view showing limited anatomical detail. Intracardiac mass not discernable. Abbreviations: RA-right atrium, RV-right ventricle, LA-left atrium, LV-left ventricle, DA-descending aorta, L-liver. **c:** Pre-operative, CT chest without contrast, coronal view showing limited mediastinal detail, left atrium myxoma is not apparent or noticeable. Abbreviations: RA-right atrium, AO-Ascending aorta, PT-pulmonary trunk, LV-left ventricle

From the patient’s perspective, on follow up, the patient was satisfied with her decision to undergo surgery to excise the cardiac myxoma. Prior to surgery, she was quite symptomatic with dyspnea on exertion. Post surgically, her physical activity levels have increased and overall she is doing much better. The recovery, however, took longer than anticipated. Her initial planned leave of absence from work was 3 months which was actually extended to 5 months. The protracted time was due to a personal family situation and findings on follow up echo of a shunt concerning for a defect in the repaired atrial septum. A transesophageal echocardiogram (TEE) was performed which did not find a defect in the septum but did confirm a thin septum and presence of mild mitral valve regurgitation. Cardiac myxomas are a risk factor for valvular heart disease as the myxoma can push violently past the mitral valve during the cardiac cycle [3]. Indeed, another surgical review study found that concurrent mitral valve surgery was the most common procedure during myxoma resection [5]. Other valvular complications due to myxoma include degenerative mitral insufficiency, mitral annular enlargement, leaflet fibrotic change and myxoma-related mitral valve lesions [5]. Fortunately, in this case, the patient did not require corrective valve surgery. Interestingly, one aspect of the workup that did affect clinical management was the lack of availability of a diagnostic CT study of the chest. The patient stated that due to insurance reasons, she was unable to obtain a chest CT earlier in the diagnostic workup with Pulmonology and Infectious Disease. A chest CT with intravenous contrast would have been able to identify the atrial mass, which in turn, would have prompted a diagnostic cardiac echo as the next step in the workup [6]. Unfortunately, in this case, the initial CT of the chest was obtained without intravenous contrast thereby limiting the diagnostic utility of the study (Fig. 4b, c) and delaying the diagnosis.

## Discussion and conclusion

### Diagnosis

**Fig. 5:**
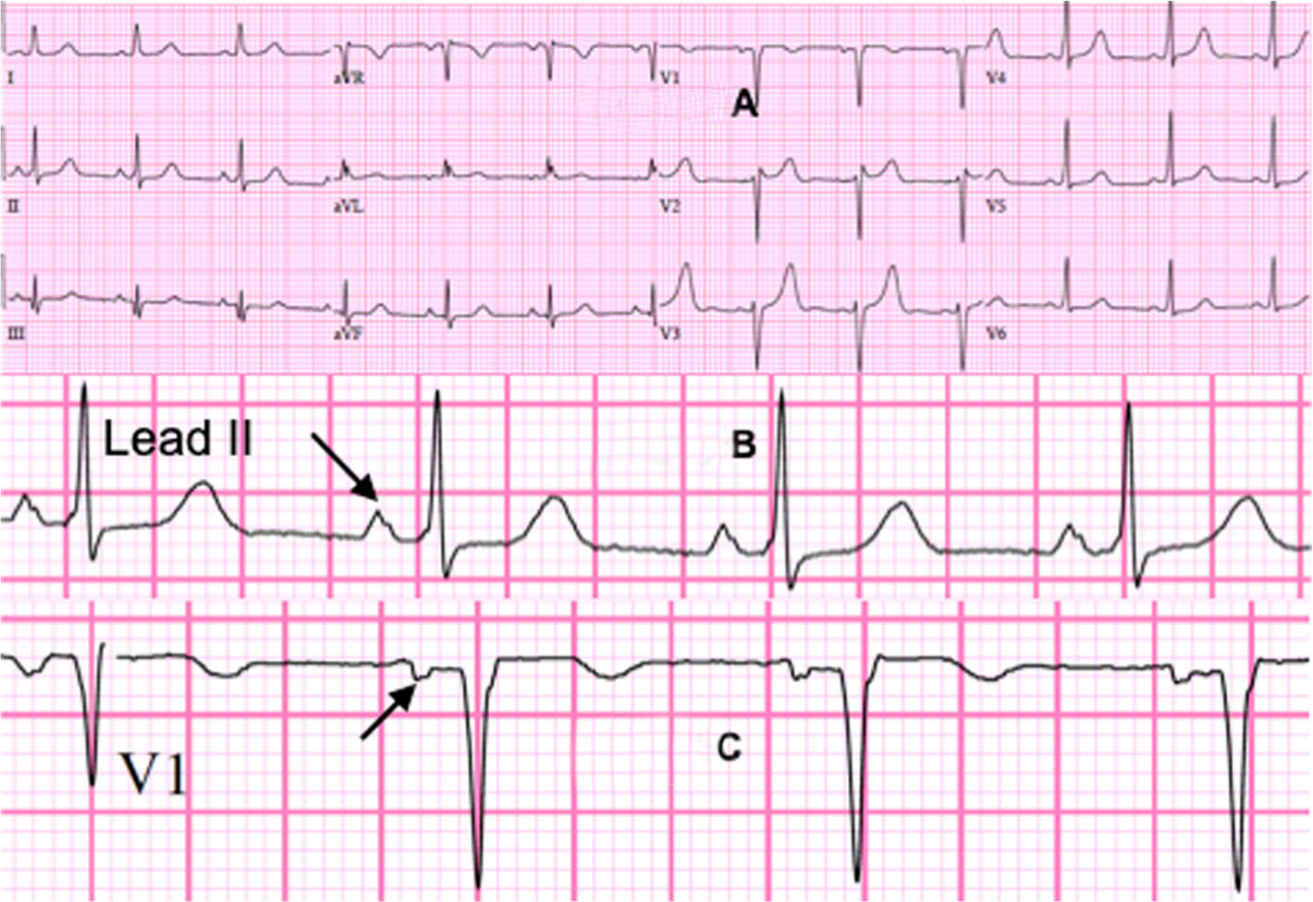
**a:** Pre-operative Electrocardiogram showing normal sinus rhythm, no ischemic changes or ventricular hypertrophy in all leads. **b:** Pre-operative Electrocardiogram, enlarged view of Lead II showing P mitrale, left atrial enlargement (arrow). **c:** Pre-operative Electrocardiogram, Lead V1 shows and deepening of the terminal negative portion of the P wave, possible left atrial enlargement (arrow)

**Fig. 6:**
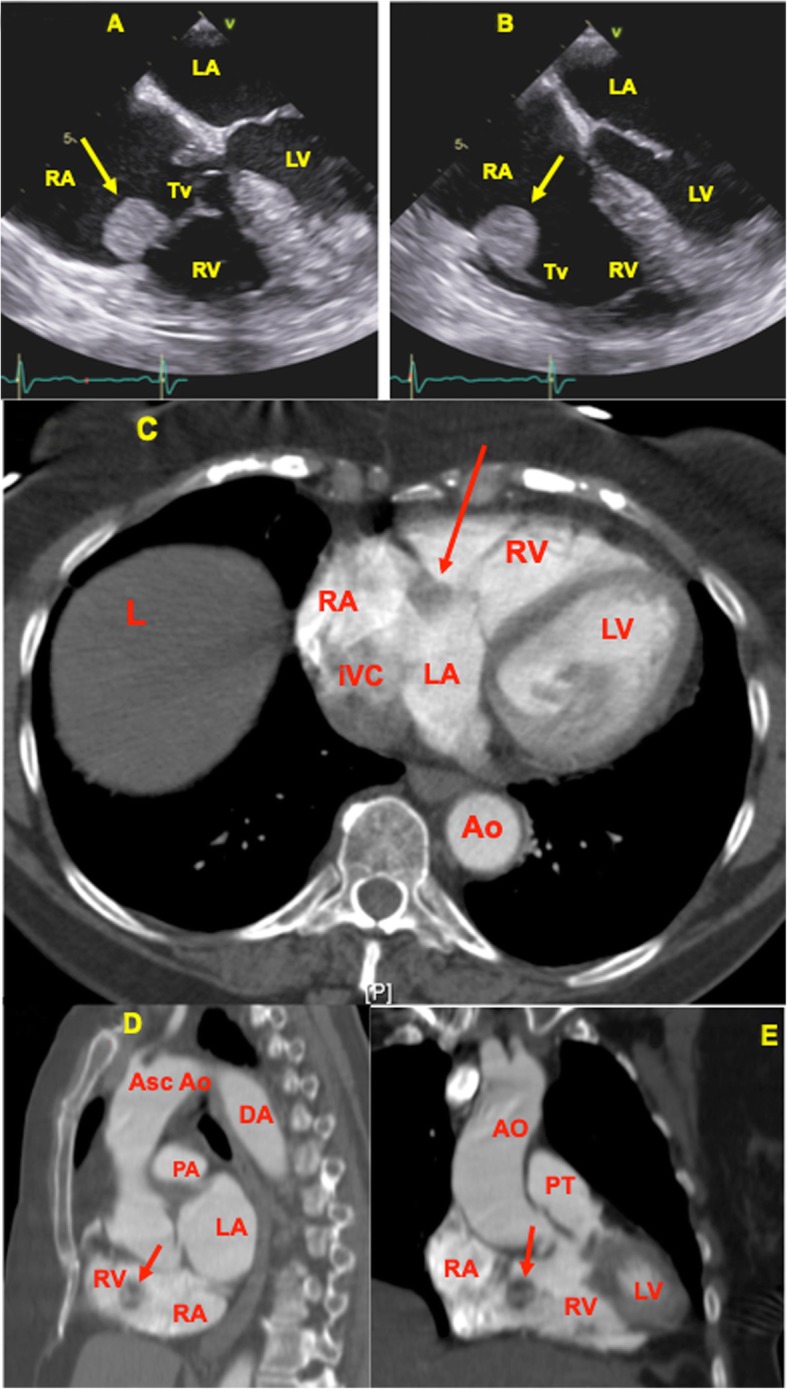
**a:** Transesophageal echocardiogram midesophageal four chamber view showing a 2.3 × 1.9 × 0.5 cm tricuspid mass (arrow), during systole. Abbreviations: RA-right atrium, RV-right ventricle, LA-left atrium, LV-left ventricle, Tv-tricuspid valve. **b**: Transesophageal echocardiogram midesophageal four chamber view showing partial obstruction of right ventricular inflow during diastole (arrow). Abbreviations: RA-right atrium, RV-right ventricle, LA-left atrium, LV-left ventricle, Tv- tricuspid valve. **c:** CT chest with contrast showing contrast enhanced axial CT scan with minimal enhancement of the mass (arrow) at the level of the tricuspid valve. The radiologist reported the finding as a 2.1 cm oval fatty mass seen along the tricuspid valve. Cardiac tumors are usually low attenuating. Abbreviations: RA-right atrium, RV-right ventricle, LA-left atrium, LV-left ventricle, Ao-aorta, IVC-inferior vena cava, L-liver. **d:** CT chest with contrast showing contrast enhanced sagittal CT scan with minimal enhancement of the mass (arrow). Cardiac tumors are usually low attenuating. Abbreviations: RA-right atrium, RV-right ventricle, LA-left atrium, Asc Ao-ascending aorta, PA-pulmonary artery, DA-descending aorta. **e:** CT chest with contrast showing contrast enhanced coronal CT scan with minimal enhancement of the mass (arrow). Cardiac tumors are usually low attenuating. Abbreviations: RA-right atrium, RV-right ventricle, LA-left atrium, LV-left ventricle, PT-pulmonary trunk, AO-ascending aorta

The diagnostic modality of choice is an echocardiography, which identifies the size, site, attachment, mobility, and also grossly differentiates the myxoma from vegetation or a thrombus, with transesophageal echocardiography as the preferred choice [3]. CT imaging of the chest with intravenous contrast may demonstrate the cardiac myxoma as a contrast filling defect as shown in Fig. 6c to e. For further characterization of a cardiac tumor, MRI with contrast is preferred, although CT coronary angiography can also be used to differentiate myxomas and thrombi [6]. Chest radiography may show cardiomegaly, abnormal cardiac silhouette mimicking mitral stenosis, unusual intracardiac calcification, pulmonary edema, biventricular hypertrophy with or without left atrial (LA) enlargement. In this case, the chest x-ray appeared largely normal except for possible left atrial enlargement as evidenced by a “double density” behind the right heart border (Fig. 4a). ECG may show left atrial enlargement, atrial fibrillation, atrial flutter or conduction disturbances. In this case, the ECG showed evidence of left atrial enlargement with a “P mitrale” wave pattern consisting of a notch (double hump) near its peak, best seen in lead II. Lead V1 also shows deepening of the terminal negative portion of the P wave (Fig. 5a to c). Definitive diagnosis would involve microscopic evaluation of the mass which typically shows thin-walled vasculature dispersed within a mucinous, myxoid background with perivascular eosinophilic cells, consistent with myxoma cells. Calretinin staining of the mass will also be strongly positive, confirming the presence of cardiac myxoma cells [7].

### Surgical approach

Myxomatous tumors are generally divided into solid or papillary groups based upon gross external features [8]. Tumors with smooth regular borders are classified as solid and can generally be excised in one piece. Papillary myxomas are characterized by an irregular and myxomatous exterior, which tend to be friable necessitating piecemeal removal. In this case, the solid tumor was retrieved in one piece. As described, myxoma is operated on by open heart surgery under cardiopulmonary bypass. The usual approach is via incision in the left atrium, posterior to interatrial groove to visualize the tumor [9]. However, given the large size of the myxoma and restricted access to the left atrium, decision was made to approach via right atriotomy. A bi-atrial approach can be used if the base of the myxoma is sessile or papillary. Controversy exists as some argue that radical resection with excision of a full-thickness portion of the septum has no benefit over conservative excision, particularly with sporadic myxoma [9]. Research may be helpful in characterizing radical versus conservative septal excision.

### Treatment

Embolization commonly occurs in about 35% of all left atrial myxomas, whereby it is actually the thrombus on the surface that embolizes more than the tumor itself and a smaller tumor of < 4.5 cm poses an increased risk. Embolization can occur in the lower extremities such as the iliac and femoropopliteal, viscera, spleen, adrenals, kidneys, and even the abdominal aorta [3]. In the upper extremity, cardiac myxoma is associated with approximately 10% of coronary embolizations, 20–35% of neurological complications (TIA) and 9–22% of embolic stroke [3]. Embolization occurs in about 10% of right atrial myxomas and consist of either pulmonary artery embolism (PE) or systemic embolization if there is an atrial septal defect [3].

In terms of treatment, organized and small thrombus can be treated medically with anticoagulants, thrombolysis or thrombectomy [10]. Anticoagulation with high dose intravenous heparin or low molecular weight heparin has variable resolution rates 13–59%. Recombinant tissue plasminogen activator can be used for lysis of a mobile, pedunculated LV thrombus [11]. On the other hand, a large mobile thrombus with or without a hemodynamic alteration, prior embolic events, and failed anticoagulation are an indication for surgical removal [10]. Furthermore, in the Ogren 2005 European study [12], right atrial (RA) thrombus was described in about 10% of patients with PE, and of patients with RA thrombus, 36% had PE and 6.5% of patients with PE confirmed at autopsy had RA thrombus [13].

In terms of myxomas, despite the fact that the majority of such tumors are benign, surgical resection is highly suggested to prevent complications including sudden death [3]. Surgical excision of cardiac myxoma shows curative with few recurrences at follow-up observation, which carries a low operative risk [14]. While the timing of surgery is not clear, it is not unusual for patients to die or experience a major complication while waiting given the risk of sudden cardiac death. Hence, some authors suggest surgery is urgent once it has been identified that a patient has a myxoma that is large enough to cause complete intracardiac obstruction [3, 15].

### Optimal time to surgical intervention

Although it is generally agreed that an intracardiac mass requires surgical intervention, there is no consensus in terms of the timing for the excisional surgery. For instance, in the case of the patient with the left atrial myxoma (Fig. 1a and b) presented in this case report, the time to cardiothoracic surgery was 2 weeks after diagnosis. Since she was symptomatic with fatigue, fevers, transient ischemic attack and dyspnea, her case was given a higher surgical priority. By comparison, a similar case involved a 77 years old female who presented with recurrent episodes of palpitations, hypertensive urgency, atrial fibrillation and dyspnea who was diagnosed with a tricuspid mass situated in the right atrium. Cardiac echocardiogram revealed a 2.3 × 1.9 × 0.5 cm tricuspid mass (Fig. 6a and b) that was also evident on CT of the chest with intravenous contrast. As mentioned previously, the cardiac myxoma appears as a filling defect within the contrast enhanced cardiac chamber of the CT chest (Fig. 6c to e). In this case, her surgery was delayed for 6 weeks due to planning for concurrent aortic valve replacement, mitral valve repair and tricuspid valve replacement. As shown, the time to surgical intervention for an intracardiac mass can vary widely between 2 weeks to 6 weeks, although priority is given to patients who are symptomatic or hemodynamically unstable. This variability in time to surgical intervention is also evident in literature review. For instance, the Kuroczynski 2009 study, which followed 57 patients undergoing cardiac myxoma excision surgery from January 1985 to December 2008, found that the duration of symptoms prior to surgery ranged from 6 to 1373 days, with a median of 96 days [16]. In the 2001 study by Pinede at al., the authors reviewed 112 cases of left atrial myxoma seen at one institution over a 40-year period from 1959 to 1998. They found that the time interval between onset of symptoms and surgical removal varied from 0 to 126 months with a median of 4 months. Interestingly, the median delay before surgery was 5.5 months before 1977, and 3 months after 1977, with the year of 1977 being significant for the introduction of echocardiography as a diagnostic procedure [17]. In the 2017 study by Lee et al., the authors examined 93 patients with cardiac myxoma who underwent surgery at their surgical institution from January 1986 to December 2015. They comment that 65 patients underwent elective surgery, whereas 28 patients with severe symptoms or embolic risk underwent emergency surgery. The authors did not specify the time duration prior to elective surgery [5]. As shown, there is no literature that directly studies the effect of surgical timing on patient prognosis, however, the general consensus is that prompt surgical excision must be performed after diagnosis because of the high risk of valvular obstruction or systemic embolization [8, 9, 17]. Given this variability in time to surgical intervention, more studies may contribute to optimize the presurgical management of patients diagnosed with an intracardiac mass in terms of decision to anticoagulate and management of the risks of valvular obstruction and systemic embolization. Interestingly, in the surgical examples from this institution, the patients were only on aspirin and none were placed on anticoagulation.

### Carney complex

The topic of Carney complex is also interesting in this case. Carney complex (CNC) is an autosomal dominant multiple neoplasia syndrome that includes cardiac myxoma, endocrine, cutaneous, and neural tumors [18]. Extra-cardiac manifestations of CNC include pigmented skin lesions, cutaneous myxomas, adrenal cortical disease, myxoid mammary fibroadenoma, and testicular tumors. It bears similarities to other syndromes such as McCune-Albright, Peutz-Jeghers, Cowden and neurofibromatosis [19].

Additional past medical history from the patient includes two right knee effusions performed 20 years ago, roughly age 30, and diagnosed with Pigmented villo-nodular synovitis (PVNS). The incidence of PVNS ranges around 1.8 per million, usually monoarticular, affecting large joints, with the knee being the most common site. Microscopically, the synovial membrane is characterized by inflammation and presence of hemosiderin deposits, lipid-laden macrophages, multinucleated giant cells and proliferation of fibroblasts and stromal cells [20].

The patient also had basal cell carcinoma of the right upper lip with surgery 8 years ago, at around age 40. She also had squamous cell carcinoma of the right lower abdomen also requiring excision this year, at age 49. Incidentally, patient also recalls she had a dark lentigines (liver spot) on her left upper arm as a child which resolved in adulthood.

Although PVNS, basal cell carcinoma and squamous cell carcinoma are not necessarily associated with cardiac myxomas, perhaps the patient may have a variant of Carney complex that involves dermatologic and soft tissue proliferation. Furthermore, her upper lip skin lesion may have been a cutaneous myxoma that was misdiagnosed as a basal cell carcinoma since they are both flesh colored. The squamous cell carcinoma may have been a pigmented nevus common in CNC. Given the rarity of sporadic primary cardiac myxoma, incidence of 0.5 per million population per year [18], and the history of 3 prior tumors, the patient may have a variant of a tumorous syndrome such as Carney complex. CNC has been linked to the PRKAR1A gene on chromosome 17q22–24 and is associated with a familial multiple endocrine neoplasia syndrome and pituitary somatomammo-trophic adenomas [21]. Interestingly, cardiac myxomas are seen in about 30–60% of CNC cases [18] and although recurrence rate is very rare in sporadic isolated myxomas, recurrence can be higher in the familial variety (10%), in Carney complex (21%), and in the presence of multiple myxomas (33%) [8, 17]. For this reason, it may be beneficial for the patient to undergo genetic counseling for the PRKAR1A gene [19]. It may also be interesting to research patients with CNC who are diagnosed with a cardiac myxoma as this larger sample size may provide greater opportunity to examine the perioperative management in minimizing embolic complications and sudden cardiac death.

## Summary

This case report illustrates the finding of a cardiac myxoma in a young patient causing intermitent fevers, dyspnea on exertion, and extreme fatigue. Although the patient initially presented with bronchitis and URI symptoms, further diagnostic steps eventually revealed the finding of the intracardiac mass. Clinical features of myxomas encompass a triad of arterial embolism, obstruction of intracardiac blood flow, and constitutional signs often include fatigue, fever, weight loss, and elevated CRP. This array of symptoms poses a diagnostic challenge as the differential diagnosis can include rheumatic mitral valve disease, pulmonary hypertension, pulmonary embolism, endocarditis, myocarditis and vasculitis. This patient’s episode of vertigo and presyncope may have been a transient ischemic attack associated with micro emboli from the myxoma. Similarly, the intermittent fevers were likely due to associated inflammatory cytokines such as interleukin-6 and other acute phase reactants such as CRP. In cases of diagnostic uncertainty involving symptoms of dyspnea on exertion, fatigue and fevers, a chest CT with contrast enhancement has clear benefits over chest CT without contrast. If the patient’s renal function presents a limitation in using contrast, then a nephrology consult may be appropriate. Cardiac echocardiography can then be used to confirm the diagnosis with subsequent cardiothoracic surgical evaluation. Although surgical resection is curative and recurrence rate is low, patients may benefit from serial echocardiograms in the outpatient setting.

## Data Availability

Not applicable, this case report does not contain any data.

## Abbreviations

CNC: Carney complex
CRP: C-reactive protein
CT: Computed tomography
CTA: Computed tomography angiography
ECG: Electrocardiogram
LA: Left atrial
LVEF: Left ventricular ejection fraction
MRI: Magnetic resonance imaging
PE: Pulmonary embolism
PVNS: Pigmented villo-nodular synovitis
RA: Right atrial
SOB: Shortness of breath
STEMI: ST-elevation myocardial infarction
TEE: Transesophageal echocardiogram
TIA: Transient ischemic attack
TTE: Transthoracic echocardiogram
URI: Upper respiratory infection
